# Bacterial vesicles block viral replication in macrophages via TLR4-TRIF-axis

**DOI:** 10.1186/s12964-023-01086-4

**Published:** 2023-03-28

**Authors:** Jeff Bierwagen, Marie Wiegand, Katrin Laakmann, Olga Danov, Hannah Limburg, Stefanie Muriel Herbel, Thomas Heimerl, Jens Dorna, Danny Jonigk, Christian Preußer, Wilhelm Bertrams, Armin Braun, Katherina Sewald, Leon N. Schulte, Stefan Bauer, Elke Pogge von Strandmann, Eva Böttcher-Friebertshäuser, Bernd Schmeck, Anna Lena Jung

**Affiliations:** 1 Institute for Lung Research, Universities of Giessen and Marburg Lung Center, German Center for Lung Research (DZL), Philipps-University Marburg, Marburg, Germany; 2 Fraunhofer Institute for Toxicology and Experimental Medicine ITEM, Biomedical Research in Endstage and Obstructive Lung Disease Hannover (BREATH), German Center for Lung Research (DZL), Member of Fraunhofer International Consortium for Anti-Infective Research (iCAIR), Hannover, Germany; 3 Institute of Virology, Philipps-University Marburg, Marburg, Germany; 4 Center for Synthetic Microbiology (SYNMIKRO), Philipps-University Marburg, Marburg, Germany; 5 Institute for Immunology, Philipps-University Marburg, Marburg, Germany; 6 Institute of Pathology, Hannover Medical School, Biomedical Research in Endstage and Obstructive Lung Disease Hannover (BREATH), German Center for Lung Research (DZL), Hannover Medical School, Hannover, Germany; 7 Institute for Tumor Immunology and Core Facility – Extracellular Vesicles, Philipps-University Marburg, Marburg, Germany; 8 Core Facility Flow Cytometry – Bacterial Vesicles, Philipps-University Marburg, Marburg, Germany; 9 Department of Pulmonary and Critical Care Medicine, Philipps-University Marburg, Marburg, Germany; 10 Member of the German Center for Infectious Disease Research (DZIF), Marburg, Germany

**Keywords:** Extracellular vesicles, Outer membrane vesicles, Bacterial and viral co-infection, Pneumonia, Macrophage, Alveolar epithelial cell, Antiviral innate immunity

## Abstract

Gram-negative bacteria naturally secrete nano-sized outer membrane vesicles (OMVs), which are important mediators of communication and pathogenesis. OMV uptake by host cells activates TLR signalling via transported PAMPs. As important resident immune cells, alveolar macrophages are located at the air-tissue interface where they comprise the first line of defence against inhaled microorganisms and particles. To date, little is known about the interplay between alveolar macrophages and OMVs from pathogenic bacteria. The immune response to OMVs and underlying mechanisms are still elusive. Here, we investigated the response of primary human macrophages to bacterial vesicles (*Legionella* *pneumophila*, *Klebsiella* *pneumoniae*, *Escherichia coli*, *Salmonella* *enterica*, *Streptococcus* *pneumoniae*) and observed comparable NF-κB activation across all tested vesicles. In contrast, we describe differential type I IFN signalling with prolonged STAT1 phosphorylation and strong Mx1 induction, blocking influenza A virus replication only for *Klebsiella*, *E.coli* and *Salmonella* OMVs. OMV-induced antiviral effects were less pronounced for endotoxin-free *Clear coli* OMVs and Polymyxin-treated OMVs. LPS stimulation could not mimic this antiviral status, while TRIF knockout abrogated it. Importantly, supernatant from OMV-treated macrophages induced an antiviral response in alveolar epithelial cells (AEC), suggesting OMV-induced intercellular communication. Finally, results were validated in an ex vivo infection model with primary human lung tissue. In conclusion, *Klebsiella*, *E.coli* and *Salmonella* OMVs induce antiviral immunity in macrophages via TLR4-TRIF-signaling to reduce viral replication in macrophages, AECs and lung tissue. These gram-negative bacteria induce antiviral immunity in the lung through OMVs, with a potential decisive and tremendous impact on bacterial and viral coinfection outcome.

**Video Abstract**

**Supplementary Information:**

The online version contains supplementary material available at 10.1186/s12964-023-01086-4.

## Introduction

Outer membrane vesicles (OMVs) are naturally released by gram-negative bacteria, which measure up to 300 nm in diameter. Gram-positive bacteria are equally able to release membrane vesicles (MVs). As a result of their biogenesis, these lipid bilayer membrane structures contain, in addition to periplasmic contents, major components of the bacterial (outer) membrane such as lipids, proteins and, in the case of gram-negative bacteria, lipopolysaccharides (LPS) [1]. In addition to their role in bacterial communication, OMVs have been associated with pathogenic effects and the transport of virulence factors, e.g. VacA from *Helicobacter* *pylori*, Shiga toxin from *Shigella dysenteriae* or ClyA from enterohemorrhagic *Escherichia* *coli* [2]. These OMVs were shown to manipulate epithelial cell and immune responses. The presence of bacterial endotoxin on the OMV surface together with other transported pathogen-associated molecular patterns (PAMPs) make them potent stimulators for immune cells, as they can still be recognized by their respective receptors [3]. The engagement of Toll-like receptors (TLRs) and Nod-like receptors in innate immune cells can trigger the release of pro-inflammatory and immunoregulatory cytokines, including the recruitment of neutrophils or the disruption of tight junctions in epithelial cell layers [4].

One important resident immune cell type in the lung are alveolar macrophages (AMs), which reside at the air-tissue interface in the alveoli. Through ingestion of inhaled particles, AMs represent the first line of defence against microorganisms and particles by phagocytosis and degradation. Upon pathogen encounter, macrophages present antigens to adaptive immune cells and release pro-inflammatory cytokines. Thereby, they can act on type I and II alveolar epithelial cells (AECs) and other immune cells (Additional file 1: Fig. S1A). AMs can be activated by several PAMPs to induce intracellular signalling and induction of distinct gene expression patterns via diverse immune receptors [5, 6], leading to MyD88 or TRIF signalling, which both in turn induce different subsets of genes (Additional file 1: Fig. S1B). Moreover, they fulfil other functions in pulmonary homeostasis and pathogenesis, making them a central signalling hub and orchestrators in lung immunity.

Influenza viruses belong to the family of *Orthomyxoviridae* and cause upper and lower respiratory tract infections, ranging from mild to severe cases. While most seasonal influenza virus infections are self-limiting, some patients develop pneumonia and acute respiratory distress syndrome which is estimated to result in up to 650,000 annual deaths worldwide [7]. Influenza A virus (IAV) mainly infects airway epithelial cells and replicates therein. Viral infection leads to pattern recognition receptor (PRR) activation and signalling via several transcription factors, namely nuclear factor kappa B (NF-κB) and interferon regulatory factor (IRF)-3/7, which induce type I and III interferon (IFN) production. This prominent IFN response consequently induces a cellular “antiviral state” in an auto- and paracrine manner [8]. Additionally, several other cytokines and chemokines are secreted upon infection. This tightly regulated host defence cytokine network recruits immune cell populations to the site of infection and orchestrates innate and adaptive immune responses [9]. However, IAV can also infect AMs, resulting in a drastically reduced number of AMs during acute infection, which need to be re-established to resolve the infection. The activation of innate immune cells and the secretion of pro-inflammatory cytokines lead to the recruitment of additional innate cells, controlling the infection and initializing tissue repair [10, 11].

Here, we analyse the response of macrophages to bacterial extracellular vesicles, and their impact on subsequent IAV infection.

## Material and methods

### Chemicals and antibodies

Ham’s F12 medium was obtained from GE Healthcare Europe (Freiburg, Germany). RPMI-1640, DMEM, GlutaMAX, Penicillin/Streptomycin and FCS were purchased from Life Technologies (Darmstadt, Germany). PBS was acquired from Capricorn Scientific GmbH (Ebsdorfergrund, Germany). Opti-MEM was obtained from Thermo Fisher Scientific (Frankfurt, Germany). Phorbol 12-myristate 13-acetate (PMA) was supplied by Sigma-Aldrich Chemie (Munich, Germany). LPS (*Salmonella* *minnesota* R595, TLR grade) was obtained from Enzo Life Sciences (Lausen, Switzerland). Pam3CSK4 was purchased from Invivogen (San Diego, USA). JAK inhibitor Ruxolitinib was obtained from Biozol Diagnostics Vertrieb GmbH (Eching, Germany). Polymyxin B was purchased from Merck Millipore (Billerica, USA). Antibodies were obtained from Abcam (Cambridge, UK): Mx1 (ab95926), influenza nucleoprotein (9G8); Cell Signaling (Cambridge, UK): phospho-IRF-3 (Ser396)(4D4G), phospho-TBK-1 (Ser172)(D52C2), TBK-1 (61223S), phospho-STAT1 (Tyr)(58D6), STAT1 (D1K9Y), mouse anti rabbit (L27A9), IRAK-1 (4359S), phospho-p38 (Thr180/Tyr182)(9211S), p38 (9212S); Thermo Fisher Scientific: goat anti-mouse (alexa fluor 488); ProteinTech: anti-IRF-3 (66670-1) or Santa Cruz Biotechnology (Heidelberg, Germany): β-actin (C4), anti-mouse (mIgGκBP-HRP, sc-516102). All other applied chemicals were of analytical grade and acquired from commercial sources.

### Bacterial culture and OMV/MV preparation

*L. pneumophila* strain Corby wildtype was handled as previously described [12]. *K. pneumoniae* (#700721/MGH78578), *E.coli* (#25922) and *S.* *enterica* serovar Typhimurium (#14028) were obtained from American Type Culture Collection (Rockville, MD, USA). *ClearColi*™ BL21 were from BioCat GmbH (Heidelberg, Germany). *S. pneumoniae* D39 Δcps were kindly provided by Sven Hammerschmidt. Bacteria were grown on agar plates overnight (MacConkey: *Kp*, *Ec*, *Sal*; LB: *Clear coli*; blood agar plates: *Sp*) and then used to inoculate liquid media (LB: *Kp*, *Ec*, *Sal*, *Clear coli*; THY: *Sp*). Bacteria were grown until late logarithmic phase at 37°C under constant shaking (MaxQ 6000, Thermo Fisher Scientific, Karlsruhe, Germany; except for *Sp*). Bacterial cultures were then spun down three times (4,500 × g, 15 min, 4°C; Multifuge X3R, Thermo Fisher Scientific). Remaining bacteria were removed by sterile filtration through 0.22 µm pores. The supernatant was concentrated with 100 kDa molecular weight cut-off filters (Merck KGaA, Darmstadt, Germany) and the vesicles were purified either by ultracentrifugation or by size exclusion chromatography (SEC). For ultracentrifugation the supernatant was ultracentrifuged at 100,000 × g, 3 h, 4°C. After washing the obtained OMV/MV pellet with sterile PBS, ultracentrifugation was repeated and the vesicle pellet was dissolved in sterile PBS. The protein content was determined by Pierce BCA protein assay kit according to the manufacturer’s instructions (Thermo Fisher Scientific), and equal protein amounts were used for stimulation experiments. For SEC, the supernatant was concentrated to 500 µL and loaded on qEVoriginal/ 70 nm Gen 2 columns (IZON Science LTD, Lyon, France), which were pre-washed with 10 mL PBS according to the manufacturer’s protocol. Vesicles were eluted using sterile PBS and fractions of 500 µL were collected. Vesicles were eluted in fractions 7–12, which was determined by nano-flow cytometry (nFCM; NanoFCM Co., Ltd, Nottingham, UK). The pooled vesicle-containing fractions were concentrated to 200–400 µL using molecular weight cut-off filters (Merck KGaA). Vesicle concentration of all preparations was determined by nFCM and equal amounts of vesicles were used for stimulation experiments. OMV/MV preparation of both purification methods were checked for contaminating bacteria by plating and stored in aliquots at -20°C.

### nFCM

For nFCM a Nano Analyzer (NanoFCM Co., Ltd, Nottingham, UK) equipped with a 488 nm laser was calibrated with 200 nm polystyrene beads (NanoFCM Co.) with a defined concentration of 2.08 × 10^8 particles/ml and also used as a reference for particle concentration. In addition, monodisperse silica beads (NanoFCM Co.) of four different sizes were used as a size reference standard to calibrate the size of the vesicles. Freshly filtered (0.1 µm) 1 × PBS was analyzed as background signal and subtracted from the other measurements. Each distribution histogram or dot plot was derived from data collected for 1 min with a sample pressure of 1 kPa. OMV samples were diluted with filtered (0.1 µm) 1 × PBS, resulting in particle counts in the optimal range of 2,500–12,000 events. Particle concentration and size distribution were calculated using nFCM software (NF Profession V1.08).

### Transmission electron microscopy (TEM)

Carbon coated copper grids (400 mesh) were hydrophilized by glow discharging (PELCO easiGlow, Ted Pella, USA). Five µL of samples were applied onto the hydrophilized grids, and stained with 2% (w/v) uranyl acetate after a short washing step with double distilled water. Samples were analyzed with a JEOL JEM-2100 transmission electron microscope using an acceleration voltage of 120 kV. Images were acquired with a F214 FastScan CCD camera (TVIPS, Gauting, Germany).

### Cell culture

Human cell lines (THP-1, A549, BEAS2B, Calu-3, HCC827) were obtained from American Type Culture Collection. Madin-Darby Canine Kidney II cells (MDCK II) were purchased from ECACC-Sigma-Aldrich (Darmstadt, Germany). THP-1 Dual™ (Dual) and THP-1 Dual™ TRIF-KO (TRIF^−/−^) cells were acquired from Invivogen Europe (Toulouse, France). Cells were cultivated in Ham’s F12, DMEM or RPMI-1640 supplemented with 1 mM sodium pyruvate, 2 mM glutamine as well as 10% heat-inactivated FCS at cell culture conditions. THP-1 Dual™ cells were cultivated in RPMI-1640 supplemented with 1% Penicillin/Streptomycin, 2 mM glutamine, 10% heat-inactivated FCS, 100 µg/mL Normocin™ and 25 mM HEPES buffer. THP-1, Dual and TRIF^−/−^ cells were differentiated in macrophage-like cells by the addition of 20 nM PMA for 24 h.

### Cloning of *Mx1* into SparQ vector

The coding sequence of *Mx1* was generated from THP-1 cDNA by Phusion PCR using Phusion High-Fidelity DNA Polymerase (New England Biolabs, Ipswich, USA) according to the manufacturer´s instructions. An HA-tag was added by fusing the HA coding sequence to the reverse primer (sense: 5′-atcggaTTCGAAATGGTTGTTTCCGAAGTGGAC-3′, antisense: 5′-tccgatGCGGCCGCTTAAGCGTAATCTGGAACATCGTATGGGTAACCGGGGAACTGGGCAAG-3′). The PCR fragment as well as the SparQ vector (Addgene, Watertown, USA) were digested with BstbI and NotI restriction enzymes (Thermo Fisher Scientific) and ligated with T4 DNA Ligase (New England Biolabs) into the SparQ vector.

### Transfection of HEK293T cells and Lentivirus production

HEK293T cells were transfected with the SparQ vector diluted in Opti-MEM containing the sequence for *Mx1* and a GFP sequence, the viral packaging vector psPAX2 and the envelope plasmid pVSV-G (Addgene) with Lipofectamine 2000 (Thermo Fisher Scientific) according to the manufacturer´s protocol. Lentivirus was produced and virus-containing supernatant was collected every day for 72 h. Supernatant was filtered using a 0.45 µm filter and THP-1 cells were transduced (see below). Empty SparQ vector without *Mx1* sequence was used for transduction of cells to generate a corresponding control cell line (VC = vector control).

### Transduction of THP-1 cells

THP-1 cells were transduced with the lentivirus from the filtered supernatant of the HEK293T cell culture (see above). Polybrene (4 µg/mL, Sigma-Aldrich) was added to improve the transduction efficacy. Cells were incubated for up to six days. GFP positive cells were sorted by flow cytometry.

### Isolation and differentiation of BDMs

Human monocytes from healthy donors were isolated by magnetic CD14 positive selection from peripheral blood mononuclear cells and differentiated into blood-derived macrophages (BDM) in the presence of 1% human AB-serum as previously described [13].

### OMV/MV stimulation of macrophages

Primary human macrophages or differentiated THP-1 cells were incubated with purified OMVs/MVs (1 µg/mL each for vesicles purified via ultracentrifugation and a multiplicity of vesicles (MOV) of 1,000 for SEC-purified vesicles) for up to 20 h in complete media. Additional inhibitor application was performed 1 h prior to vesicle treatment. The stimulated macrophages were either used for protein or RNA isolation or subsequent infection experiments. The obtained cytokine containing supernatant was used for ELISA or LDH analysis or sterile filtered and used for epithelial cell stimulation.

### Virus purification and virus titration

Virus purification and virus titration was performed in MDCK II cells as described before [14]. The viral titer was determined by plaque assay as previously described [15]. Briefly, confluent MDCK II cells were infected with serially diluted virus or tissue supernatant (in MDCK II culture medium without FCS). Virus was allowed to adsorb to the cells for 1 h at 37°C. Then, the inoculum was replaced with fresh media (supplemented with 1 μg/mL TPCK treated trypsin (for IAV) and 1% Avicel® PH-101 (Sigma-Aldrich)). MDCK II cells were stained with crystal violet or virus-infected cells were stained with primary antibody (1:2,000) (mouse anti-influenza A nucleoprotein, MCA400, Bio-Rad, Germany) and the secondary antibody (1:4,000) (Goat Anti-Mouse IgG-HRP, 170–6516, Bio-Rad, Germany) for plaque assay. The plaques were manually counted and expressed as plaque forming units (pfu) per mL.

### Virus infection

After 20 h pre-stimulation with bacterial vesicles or cytokine containing supernatant, cells were infected with IAV or vesicular stomatitis virus (VSV). Macrophages were infected with H1N1 (A/WSN/33) or VSV, while epithelial cells were infected with H1N1 (A/Hamburg/2009/pdm) both in fresh media without FCS. After 1 h of inoculation, free virus was removed and media was replaced with TPCK-trypsin containing media for IAV or blank media for VSV. Cells were incubated for up to 48 h to allow multicyclic replication.

### Preparation of human PCLS

Lung tissue was acquired from patients who underwent lobe resection due to lung cancer at the Hannover Medical School (MHH, Hannover, Germany). Only tissue from macroscopically and microscopically disease-free parts of the lung were used for experiments. Human precision-cut lung slices (PCLS) were prepared as described before [16]. Two tissue slices per well were cultivated in a 24 well plate under submerged condition (DMEM/F12 supplemented with 1% Penicillin/Streptomycin) at 37°C, 5% CO_2_ overnight.

### IAV infection of PCLS

PCLS were stimulated with 1 µg/mL of OMVs purified from *L.* *pneumophila* or *K. pneumoniae* diluted in DMEM/F12 supplemented with 1% Penicillin/Streptomycin at 37°C, 5% CO_2_ for 20 h. The media was removed and PCLS were inoculated with 25,000 pfu/well in 250 µL of influenza virus A/California/04/2009(H1N1pdm). PCLS were incubated at 35°C and rocked every 15 min during inoculation to enable homogenous virus infection. Afterwards, the inoculum was discarded and replaced by DMEM/F12 supplemented with 1% Penicillin/Streptomycin. After incubation for 48 h, the supernatant was collected for virus detection, LDH release and cytokine quantification. Samples for the cytokine measurements were supplemented with 0.2% protease inhibitor cocktail (P1860, Sigma-Aldrich, Munich, Germany) and stored at −80°C until analysis. PCLS were frozen and stored at −80°C until RNA purification as previously described [17].

### LDH

LDH release assay was performed according to the manufacturer’s instructions using Pierce™ LDH Cytotoxicity Assay Kit obtained from Roche (Mannheim, Germany) or Cytotoxicity Detection Kit^PLUS^ (LDH) (Roche, Merck). The absorbance was measured using a microplate reader infinite F200Pro (Tecan, Männedorf, Switzerland).

### ELISA

Cytokines CXCL8/IL-8, CXCL10/IP-10 and IL-1β were quantified from cell-free supernatant using commercially available ELISA kits (R&D Systems, Wiesbaden, Germany) according to the manufacturer’s instructions.

### QUANTI-blue™ assay

For determining the reporter activity of SEAP in cell culture supernatant from THP-1 Dual™ and corresponding TRIF^−/−^ cells, QUANTI-Blue™ assay was performed according to the manufacturer’s protocol. Briefly, cell culture supernatant was collected after stimulation of cells with OMVs. First, QUANTI-Blue™ Solution and cell culture supernatant were dispensed in a flat-bottom 96-well plate. After incubation at 37°C for 150 min, optical density at 630 nm was determined in a microplate reader infinite F200Pro.

### QUANTI-luc™ assay

To determine the reporter activity of Lucia luciferase in cell culture supernatant from THP-1 Dual™ and corresponding TRIF^−/−^ cells, QUANTI-Luc™ assay was performed according to the manufacturer’s protocol. Briefly, cell culture supernatant was dispensed in a white flat-bottom 96-well plate (BRAND GmbH & Co. KG, Wertheim, Germany). QUANTI-Luc™ assay solution was added and luminescence measurement was immediately performed with a 0.1 s reading time in a microplate reader infinite F200Pro.

### Western blot

For determination of protein expression or phosphorylation, Western Blot was performed as previously described [18].

### RNA preparation and real-time PCR

For gene expression analysis, RNA isolation was carried out by phenol-chloroform extraction and reverse transcribed as previously described [12]. Quantitative real-time PCR was then performed in a ViiA7 (Thermo Fisher Scientific) with Luna Universal qPCR Master Mix (New England BioLabs) and specific primer pairs. By using the 2^−ΔΔCT^ method [19], x-fold induction was calculated and results were normalized to corresponding control cells.

18S: fwd: 5′-GACTCTTTCGAGGCCCTGTA-3′, rev: 5′-CACCAGACTTGCCCTCCAAT-3′

CXCL8: fwd: 5′-ACTGAGAGTGATTGAGAGTGGAC-3′, rev: 5′-AACCCTCTGCACCCAGTTTTC-3′

IFI44: fwd: 5′-TATCCAGACAGAGCAGCTACC-3′, rev: 5′-ATAGAGAAGGCTAAGCCGCTTC-3′

IFIT1: fwd: 5′-ATGCAGGAAGAACATGACAACC-3′, rev: 5′-TCTGGACACTCCATTCTATAGCG-3′

IFNA1: fwd: 5′-ACAGGAGGACCTTGATGCTC-3′, rev: 5′-TCTGCTGGATCAGCTCATGG-3′

IFNB: fwd: 5′-AACATGACCAACAAGTGTCTCC-3′, rev: 5′-TGTCCTTGAGGCAGTATTCAAG-3′

IL1B: fwd: 5′-AGCTCGCCAGTGAAATGATGG-3′, rev: 5′-CAGGTCCTGGAAGGAGCACTTC-3′

IL12B: fwd: 5′-GCCCAGAGCAAGATGTGTCA-3′, rev: 5′-CACCATTTCTCCAGGGGCAT-3′

Mx1: fwd: 5′-GGGCTTTGGAATTCTGTGGC-3′, rev: 5′-CCTTGGAATGGTGGCTGGAT-3′

NP: fwd: 5’-GAAATTTCAAACAGCTGCACAAAG-3′, rev: 5′-AATATGAGTGCAGACCGTGC-3′

RPS18: fwd: 5’-GCGGCGGAAAATAGCCTTTG-3′, rev: 5′-GATCACACGTTCCACCTCATC-3′

### Immunofluorescence

THP-1 cells were differentiated on coverslips by addition of 20 nM PMA and incubated with OMVs/MVs for 20 h. Cells were subsequently infected with H1N1 (A/WSN/33; multiplicity of infection (MOI) 0.1) for 4 h. After 15 min fixation with 3% paraformaldehyde, slides were washed three times with PBS and permeabilized with 0.2% triton X-100 in TBS (10 min, room temperature). After blocking (1% BSA in TBS/0.2% triton X-100), cells were incubated with α-NP antibody (1:250, in blocking solution). Secondary antibody (1:1000) together with DAPI (1:2000) was incubated for 1 h in the dark. Mounted coverslips were analyzed on a fluorescence microscope (AxioVision, Zeiss, Jena, Germany).

### Ethical statement

Experiments with human lung tissue slices were approved by the ethics committee of the Hannover Medical School (MHH, Hannover, Germany) and are in compliance with *The Code of Ethics of the World Medical Association* (number 2701–2015). All patients or their next of kin gave written informed consent for the use of lung tissue for research. All blood donors gave informed written consent (Ethics approval number: 161/17).

### Statistics

Data are shown as mean values + SEM for at least three biologically independent experiments. Prism 6.07 (GraphPad, La Jolla, USA) was used. The one- or two-way ANOVA tests were performed for unpaired samples. P-values ≤ 0.05 were considered statistically significant. If not indicated otherwise, tests were performed vs. corresponding control (*).

### Availability of data and materials

All data generated or analysed during this study are included in this article and its supplementary file.

## Results

### Pro-inflammatory activation of macrophages by OMVs/MVs

**Fig. 1:**
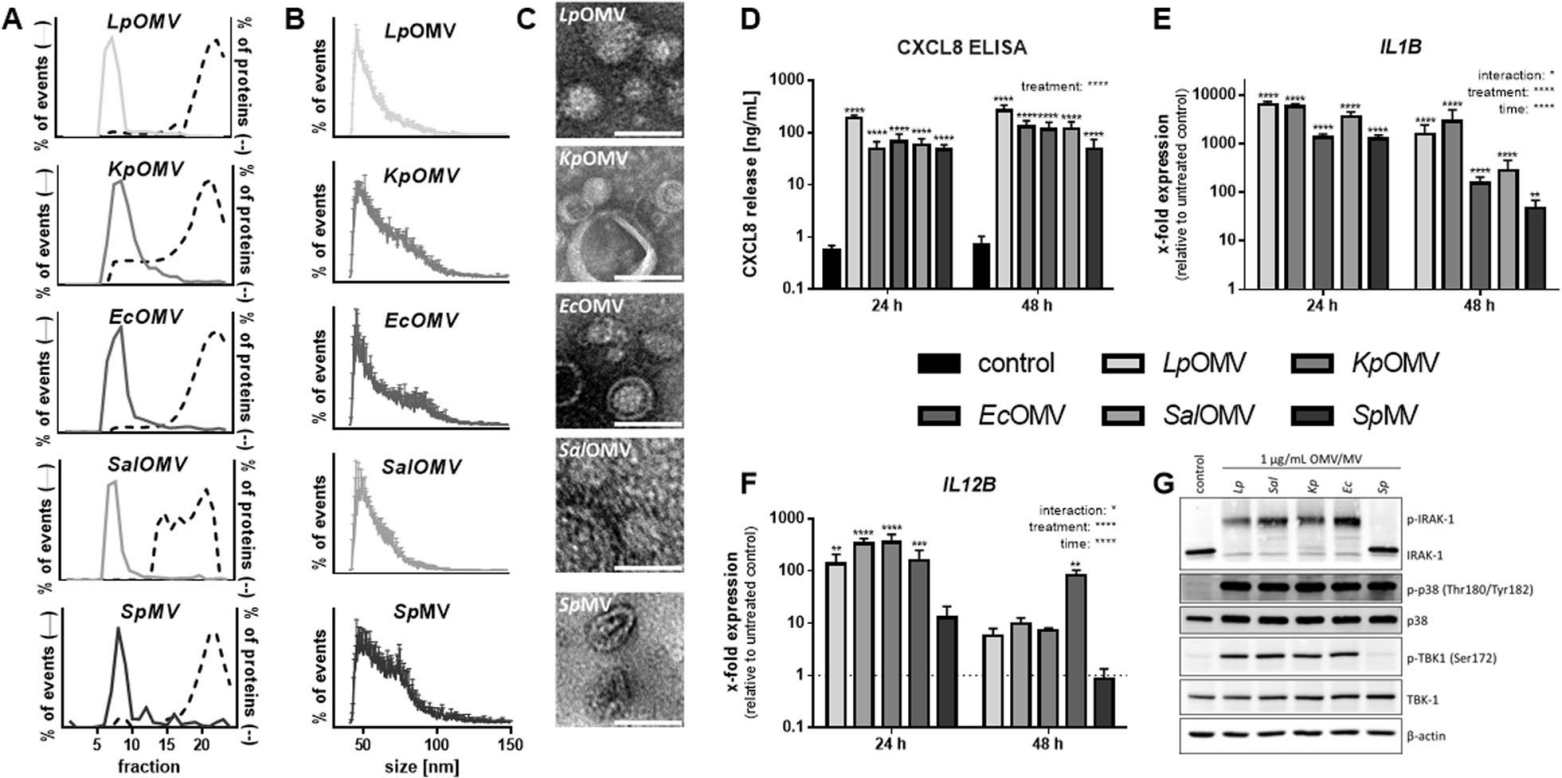
Characterization of bacterial vesicles and response in human macrophages. **A** Separation of bacterial extracellular vesicles from free proteins via size exclusion chromatography from different bacterial supernatants (*Legionella* *pneumophila* (*Lp*), *Klebsiella* *pneumoniae* (*Kp*), *Escherichia* *coli* (*Ec*), *Salmonella* *enterica* serovar Typhimurium (*Sal*), and *Streptococcus* *pneumoniae* (*Sp*)). Vesicle concentration in each fraction was determined by nano-flow cytometry (nFCM) and proteins were quantified by BCA. **B** Vesicle size distributions of purified OMVs/MVs were determined by nFCM. **C** TEM images of purified OMVs/MVs. Scale bar = 50 nm. **D–G** BDMs were stimulated with OMVs/MVs (1 µg/mL each) from different bacteria or left untreated for control for up to 48 h. **D** CXCL8 release was determined by ELISA and is depicted in ng/mL. Expression of *IL1B* (**E**) and *IL12B* (**F**) were determined by qPCR, results are normalized to *RPS18* and are depicted relative to untreated control cells. **G** After 1 h incubation with bacterial vesicles, expression and phosphorylation of IRAK-1, p38 and TBK-1 were determined by Western Blot. Representative results of four biological independent replicates are shown. Bars represent mean values + SEM from three (**B**) to four (**D–F**) independent experiments. Statistics: 2-way ANOVA (D-F); *p < 0.05, **p < 0.01, ***p < 0.001, ****p < 0.0001; ns = not significant; n = 3–4

To test the innate immune response of human primary blood-derived macrophages (BDM) to OMVs/MVs from different bacteria, vesicles from *Legionella* *pneumophila* (*Lp*), *Klebsiella* *pneumoniae* (*Kp*), *Escherichia* *coli* (*Ec*), *Salmonella* *enterica* serovar Typhimurium (*Sal*) and *Streptococcus* *pneumoniae* (*Sp*) were isolated via size exclusion chromatography (SEC). Fractions were analysed for the amount of vesicles by nano-flow cytometry (nFCM) and for the presence of proteins by BCA (Fig. 1A). Vesicles from fractions 7–12 were pooled and used for experiments. OMVs/MVs had a comparable size distribution profile (Fig. 1B), did not differ in average size (Additional file 1: Fig. S2A), were concentrated equally (Additional file 1: Fig. S2B) and were visualized by transmission electron microscopy (TEM; Fig. 1C). BDMs were stimulated with OMVs/MVs for up to 48 h, corresponding to equal vesicle concentrations of the different bacteria (Additional file 1: Fig. S2C). Incubation of BDMs with vesicles was not cytotoxic (Additional file 1: Fig. S2D).

OMVs/MVs broadly induced CXCL8 expression and release from macrophages (Additional file 1: Fig. S3A and 1D), while *IL1B* and *IL12B* mRNA induction was time- and species-dependent (Fig. 1E + F). The release of IL-1β was dependent on the species the bacterial vesicles were isolated from (Additional file 1: Fig. S3B). Although phosphorylation of p38 was comparable in BDMs after 1 h of vesicle incubation, only OMVs from gram-negative bacteria induced degradation of IRAK-1 and phosphorylation of TBK-1 but not MVs from gram-positive (Fig. 1G and Additional file 1: S4A-D).

**Fig. 2:**
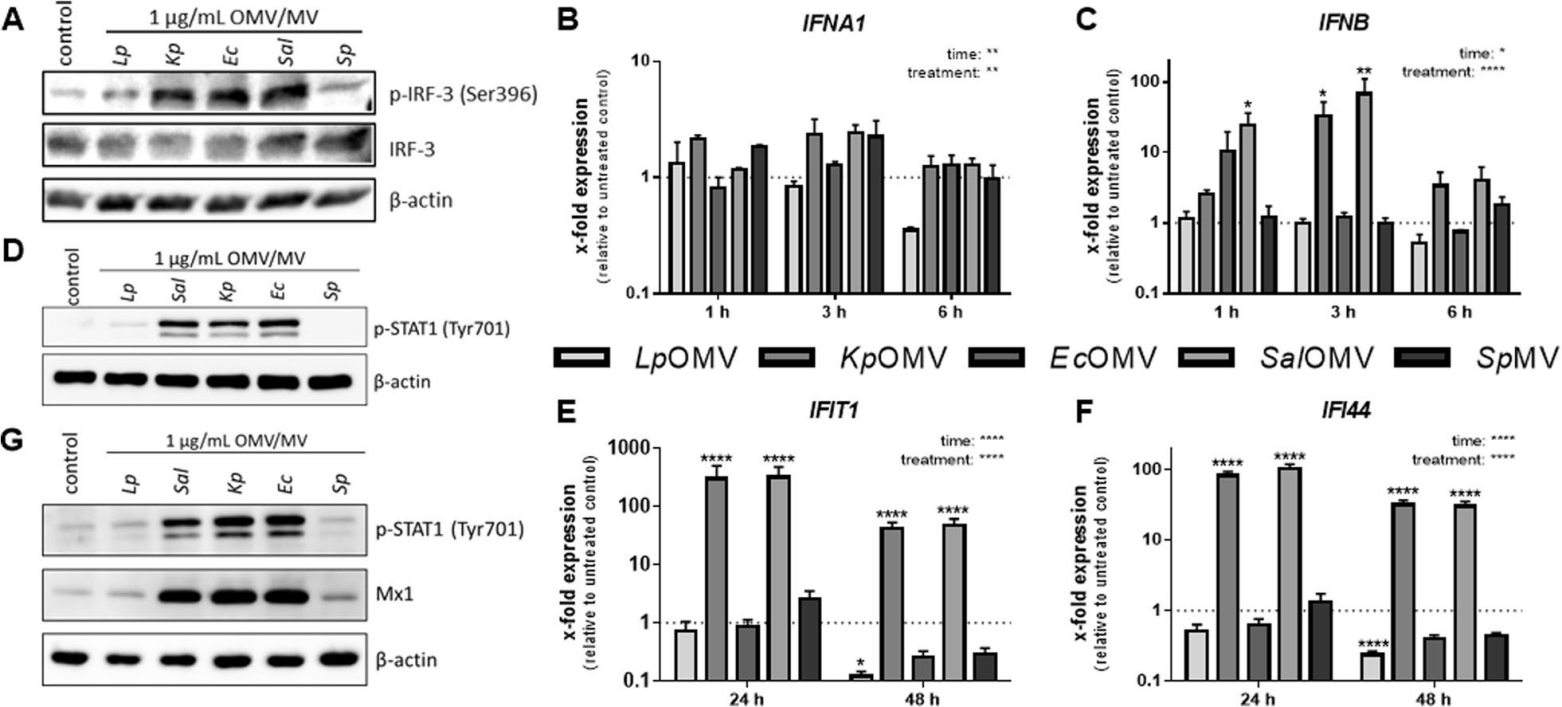
Bacterial extracellular vesicles activate type I interferon response in BDMs. BDMs were stimulated with OMVs/MVs (1 µg/mL each) from different bacteria or left untreated as control. **A** Phosphorylation and expression of IRF-3 was determined after 1 h of OMV/MV incubation by Western Blot. Representative result of three biological independent replicates is shown. Expression of *IFNA1* (**B**), *IFNB* (**C**), *IFIT1* (**E**) and *IFI44* (**F**) was measured by qPCR, results are normalized to *RPS18* and are depicted relative to untreated control cells. **D** Phosphorylation of STAT1 was determined by Western Blot after 2 h of OMV/MV stimulation. Representative result of four biological independent replicates is shown. **G** Phosphorylation of STAT1 and expression of Mx1 were determined by Western Blot after 20 h of bacterial vesicle stimulation. Representative results of four biological independent replicates are depicted. Bars represent mean values + SEM from four independent experiments. Statistics: 2-way ANOVA; *p < 0.05, **p < 0.01, ****p < 0.0001; n = 4

Besides the activation of NF-κB and AP-1 and their downstream targets, PRR signalling can lead to activation of IRFs. Thus, phosphorylation of IRF-3 was determined. *Kp*/*Ec*/*Sal*OMVs increased phosphorylation of IRF-3 in BDMs (Fig. 2A), resulting in the expression of IFN I (Fig. 2B + C), downstream phosphorylation of STAT1 (Fig. 2D and Additional file 1: S4E) and induction of interferon-stimulated genes (ISGs; *IFIT1*, *IFI44* and Mx1; Fig. 2E-G). As phosphorylation events are typically short-lived and known to play a critical role in switching immune signalling cascades on and off, phosphorylation of STAT1 was also examined 20 h after addition of *Kp*/*Ec*/*Sal*OMVs (Fig. 2G and Additional file 1: S4F + G). The experiments showed that OMVs are not only activating pro-inflammatory signalling in macrophages, but also antiviral signalling is induced.

### OMV pre-incubation alters IAV replication in macrophages

**Fig. 3:**
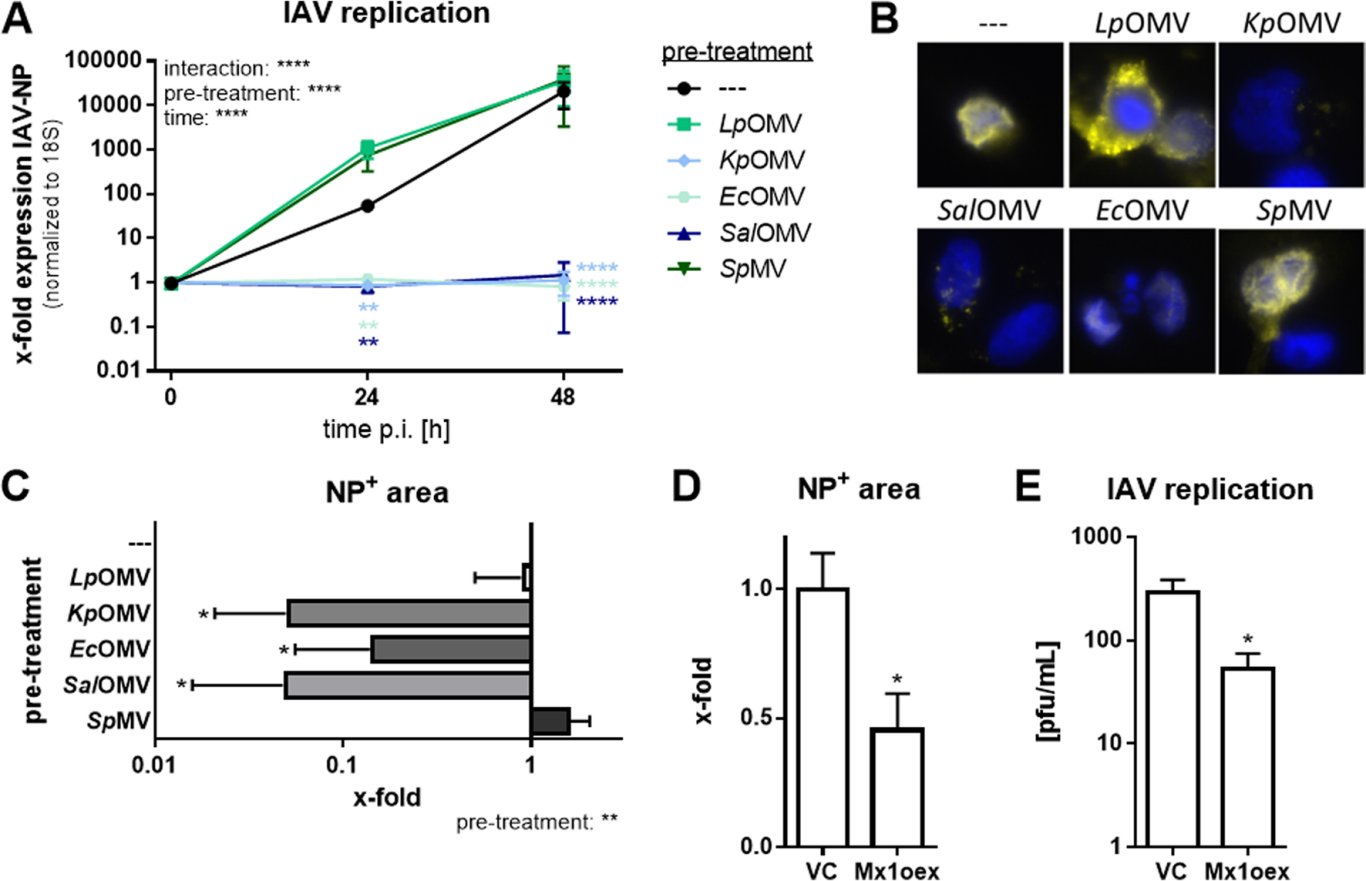
Mx1-inducing OMVs block influenza A virus replication in THP-1 cells. **A** Influenza A virus replication in differentiated THP-1 cells. Cells were either pre-treated with OMVs/MVs (1 µg/mL each), or left untreated for control (–). After 20 h pre-treatment, cells were infected with A/WSN/33(H1N1) (MOI 0.001) for 24 and 48 h. IAV replication was determined by qPCR against IAV-NP normalized to 18S. Mean values ± SEM of three to five independent experiments are shown. **B** Differentiated THP-1 cells were pre-treated with OMVs/MVs (1 µg/mL each) or left untreated for control (–). After 20 h pre-incubation, cells were infected with A/WSN/33(H1N1) (MOI 0.1) for 4 h. After fixation, cells were stained with an α-influenza NP antibody (yellow) and DAPI (blue). A representative result from four biological independent experiments is shown. **C** Quantification of NP positive (NP^+^) area from (**B**). Bars represent mean values of four independent experiments + SEM. **D** THP-1 cells stably overexpressing Mx1 (Mx1oex) and empty vector control (VC) cells were infected with influenza virus A/WSN/33(H1N1) (MOI 0.1) for 4 h. After fixation and immunofluorescence staining with α-influenza NP, the NP^+^ area was quantified and is depicted relative to VC cells. Bars show mean values of four independent experiments + SEM. **E** Mx1oex and VC cells were infected with A/WSN/33(H1N1) (MOI 0.001) for 6 h. Viral replication was determined by plaque assay and results are depicted as plaque forming units (pfu) per mL. Bars represent mean values + SEM of four independent experiments. Statistics: 2-way ANOVA (**A**), 1-way ANOVA (**C**), unpaired t-test (**D** + **E**); *p < 0.05, **p < 0.01, ****p < 0.0001; n = 3–5

Mx1 is a well-known antiviral factor inhibiting IAV replication [20], which was expressed upon *Kp*/*Ec*/*Sal*OMV treatment of macrophages. We therefore hypothesized that OMV pre-treatment alters viral replication. IAV infection experiments were set up in THP-1 cells, which express similar levels of Mx1 upon *Kp*/*Ec*/*Sal*OMV treatment on mRNA and protein level (Additional file 1: Fig. S5A + B) and induce the phosphorylation of STAT1, while total STAT1 protein remains stable (Additional file 1: Fig. S5C), with an H1N1 strain (A/WSN/33) that infects and replicates in macrophages [21]. Pre-stimulation of THP-1 cells with *Lp*OMV/*Sp*MV increased IAV replication 24 h post infection (p.i.) compared to infected control (–-), whereas pre-treatment with Mx1-inducing OMVs (*Kp*/*Ec*/*Sal*) blocked IAV replication (Fig. 3A). While TLR2/1 agonist Pam3CSK4 pre-treatment could mimic the effect observed with *Lp*OMV/*Sp*MV, which are both known to signal via TLR2/1 [12, 22, 23], soluble LPS as a TLR4 agonist did not reproduce the *Kp*/*Ec*/*Sal*OMV effect (Additional file 1: Fig. S5D). Differences in IAV load after pre-treatment could also be observed by immunofluorescence staining against the viral nucleoprotein (NP) (Fig. 3B). *Kp*/*Ec*/*Sal*OMV pre-treatment blocked IAV replication and the NP-positive area was significantly reduced (Fig. 3C). This could not be observed after *Lp*OMV/*Sp*MV pre-treatment. To investigate if Mx1 induction is sufficient for the observed blocked IAV replication, Mx1 was stably overexpressed (oex) in THP-1 macrophages to mimic OMV pre-stimulation (Additional file 1: Fig. S5E). IAV infection of Mx1oex cells resulted in a reduction of immunofluorescent NP-positive area 4 h p.i. (Fig. 3D) and in a reduced viral replication 6 h p.i. compared to an empty vector control (VC) (Fig. 3E) although to a lesser extent compared with OMV pre-stimulation (Fig. 3A).

**Fig. 4:**
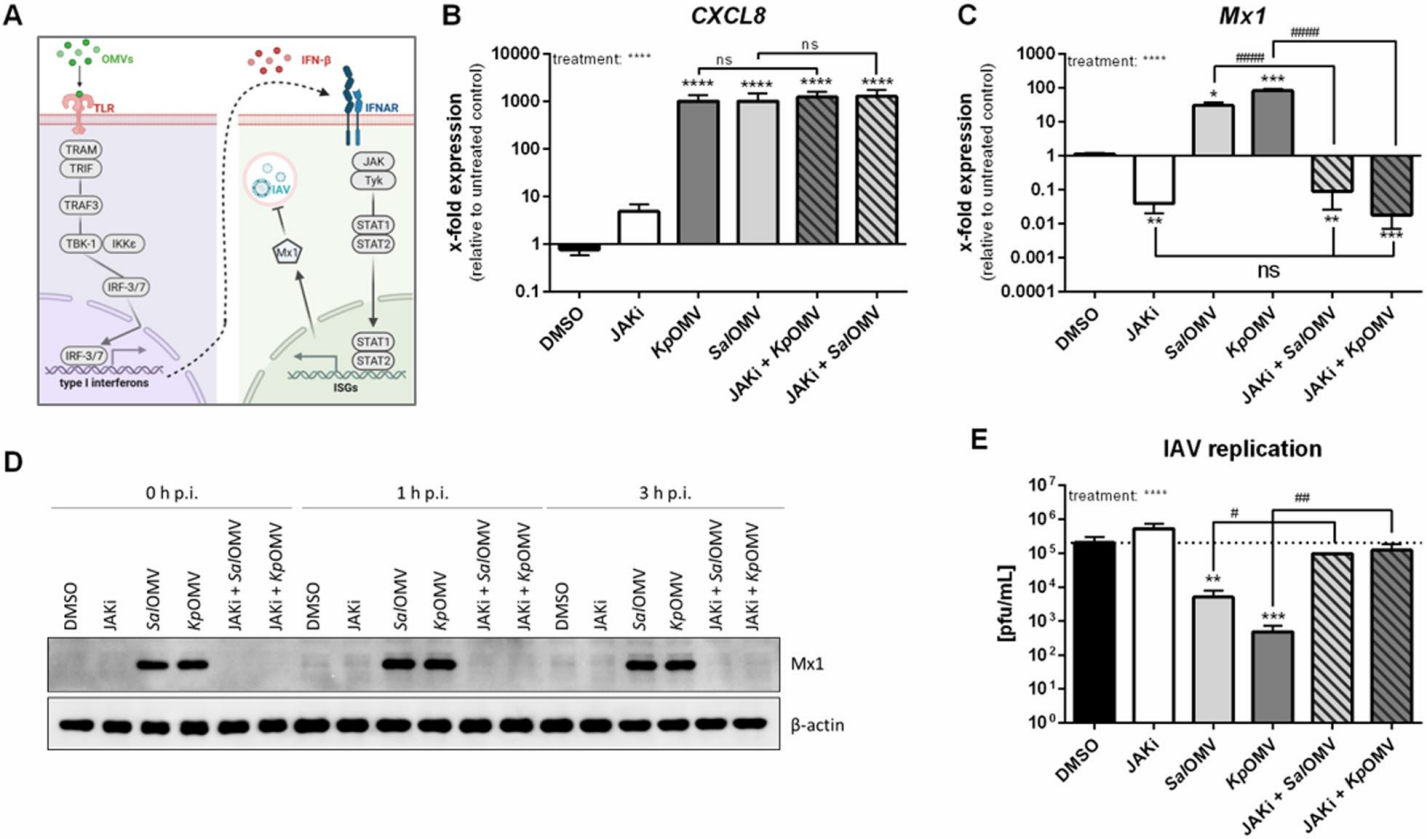
Inhibition of JAK-signalling rescues influenza A virus replication in macrophages. **A** OMVs activate TRIF-IRF-signalling in macrophages, which in turn induces *IFN-*β expression and release with subsequent IFNAR-signalling. IFNAR signals via JAK/STAT and induces the expression of ISGs, one of which is Mx1. This leads to a block of IAV replication. **B**–**E** THP-1 cells were pre-incubated for 1 h with 10 µM JAK inhibitor (JAKi) before addition of OMVs (1 µg/mL; *Kp*/*Sal*) for 20 h. **B–C** *CXCL8* (B) and *Mx1* (**C**) expression was determined by qPCR and results are normalized to *RPS18* and depicted relative to untreated control. Bars show mean values of three to four independent experiments + SEM. **D** + **E** OMV pre-treated cells were additionally infected with IAV (MOI 0.001). **D** Western blot shows Mx1 protein expression at 0–3 h post infection (p.i.). **E** Viral replication was determined by plaque assay 24 h after infection. Bars show mean values of four independent experiments + SEM. Statistics: 1-way ANOVA (**B** + **C** + **E**); *p < 0.05, **p < 0.01, ***p < 0.001, ****p < 0.0001; *compared to DMSO control, # as depicted in the graph; ns = not significant; n = 4

In conclusion, *Kp*/*Ec*/*Sal*OMV activated macrophages in a classical pro-inflammatory manner and induced antiviral genes resulting in activation of IFN-α/β receptor (IFNAR), downstream phosphorylation of STAT1 and expression of Mx1, which can directly interfere with IAV replication (Fig. 4A). To confirm the importance of JAK/STAT signalling for the observed induction of an antiviral response, a JAK inhibitor (JAKi) was applied before vesicle stimulation. JAK inhibition did not change *Kp*/*Sal*OMV-induced *CXCL8* expression (Fig. 4B), but significantly reduced *Mx1* on mRNA (Fig. 4C) and protein level even upon IAV infection (Fig. 4D and Additional file 1: S6A). Accordingly, JAKi reversed OMV-induced viral replication blockade (Fig. 4E).

### Antiviral response upon OMVs is TLR4-TRIF-dependent

**Fig. 5:**
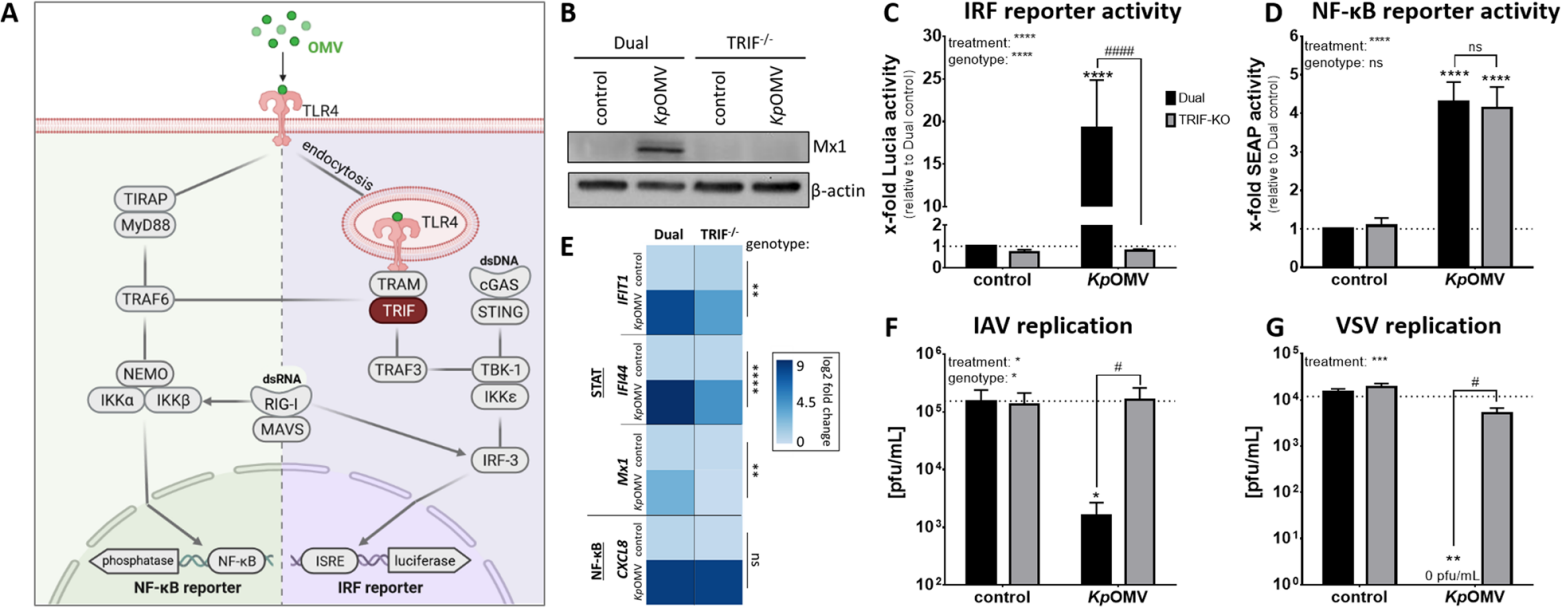
OMVs induce antiviral signalling in macrophages via TRIF. **A** THP-1 Dual reporter cells induce the expression of secreted embryonic alkaline phosphatase (SEAP) after activation of the NF-κB pathway and the expression and secretion of Lucia luciferase upon activation of the IRF pathway. Additionally, TRIF^−/−^ cells have a stable knockout of the adapter molecule TRIF. (**B-F**) THP-1 reporter cells (= Dual; black bars) and TRIF^−/−^ cells (grey bars) were differentiated and subsequently stimulated with *Kp*OMV (1 µg/mL) for 20 h or left untreated for control. After the indicated time, supernatant, RNA and/or proteins were collected. **B** Representative Western Blot image of Mx1 protein expression. **C** + **D** Lucia reporter activity (**C**) and SEAP reporter activity (**D**) was determined in cell culture supernatant. The same supernatant was used to determine the activity of both reporters. **E** Relative mRNA expression for STAT- and NF-κB-dependent target genes (from top to bottom: *IFIT1*, *IFI44*, *Mx1*, *CXCL8*) was determined by qPCR and results are normalized to *RPS18* and depicted relative to unstimulated Dual control. Fold changes were log2 transformed. Cells were additionally infected with A/WSN/33(H1N1) (MOI 0.1) for 24 h (**F**) or VSV (MOI 0.1) for 12 h (**G**). Viral replication was determined by plaque assay. Bars show mean values of four (**C–F**) to five (**G**) independent experiments + SEM. Statistics: 2-way ANOVA (**C–G**); *p < 0.05, **p < 0.01, ****p < 0.0001; * compared to unstimulated Dual control, # as depicted in the graph; ns = not significant; n = 4–5

To identify the involved PRR, commercial immune agonists were applied alone or in combination with LPS to mimic OMVs. However, none mirrored Mx1 induction observed with *Kp*OMVs (Additional file 1: Fig. S7), suggesting a differential and prolonged activation of macrophages by OMVs due to their ligand composition or spatial presentation to PRRs. The involved immune receptors were further assessed by THP-1 reporter cells for NF-κB- and IRF-signalling (Fig. 5A). Upon *Kp*OMV treatment, THP-1 cells showed significantly increased expression of Mx1 protein (Fig. 5B and Additional file 1: S6B), which was lost in TRIF deficient (TRIF^−/−^) THP-1 cells (Fig. 5B and Additional file 1: S6B). The same was observed for the IRF reporter (Fig. 5C), while NF-κB reporter activation and *CXCL8* induction were high in both THP-1 and TRIF^−/−^ cells upon stimulation (Fig. 5D + E). STAT-dependent *IFIT1*, *IFI44* and *Mx1* gene expression was induced upon *Kp*OMV stimulation in THP-1 reporter cells (Fig. 5E), while it was significantly reduced in equally stimulated TRIF^−/−^ cells (Fig. 5E). Additional IAV infection after pre-stimulation resulted in decreased viral replication, whereas TRIF deletion rescued IAV replication (Fig. 5F). To investigate whether *Kp*OMVs elicit a general antiviral response in macrophages, cells were infected with vesicular stomatitis virus (VSV), which replicated in control cells, but was blocked in *Kp*OMV pre-treated cells and could be rescued by TRIF deletion as well (Fig. 5G).

**Fig. 6:**
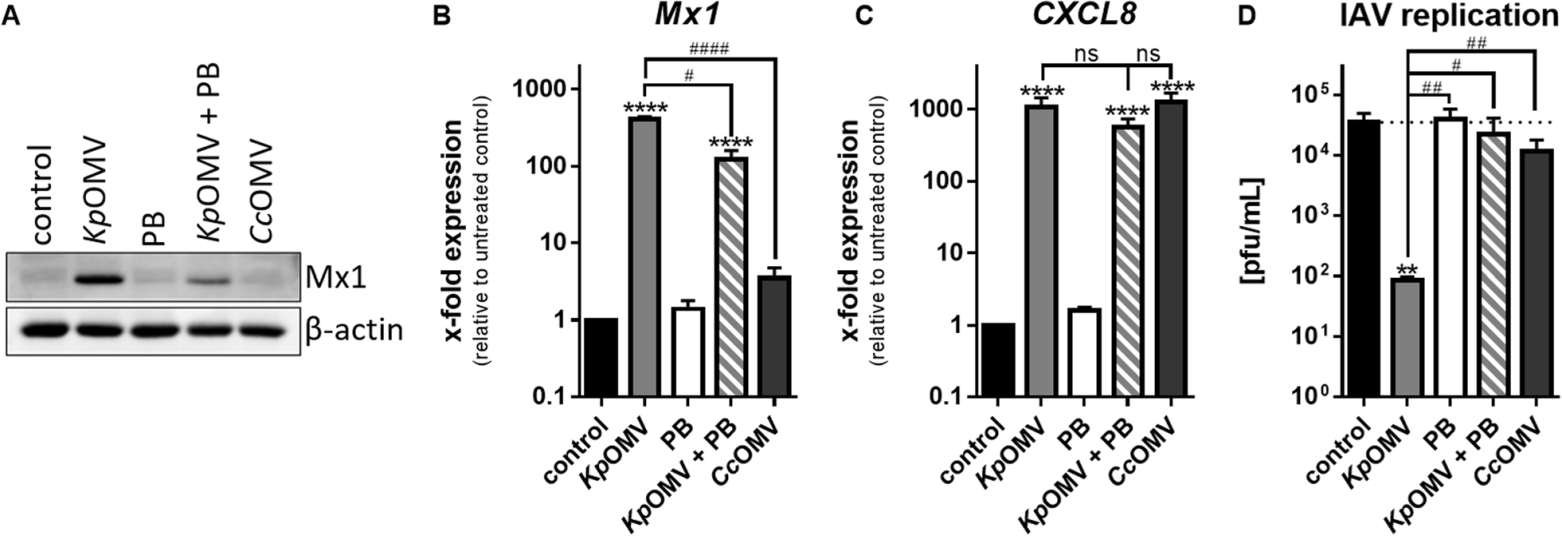
OMV-induced Mx1 expression is lost after LPS inhibition. THP-1 cells were incubated for 20 h with OMVs (1 µg/mL; *Kp* or *Clear coli* (*Cc*)) alone or in combination with 20 µg/mL Polymyxin B (PB) or left untreated for control. **A** Mx1 protein expression was determined by Western Blot. A representative result of three biological independent experiments is shown. *Mx1* **B** and *CXCL8* **C** expression was determined by qPCR. Bars show mean values of four independent experiments + SEM. **D** THP-1 cells were incubated for 20 h with OMVs (1 µg/mL; *Kp* or *Cc*) alone or in combination with 20 µg/mL PB or left untreated for control and then additionally infected with A/WSN/33(H1N1) (MOI 0.001). Virus replication was determined by plaque assay 24 h after infection. Bars show mean values of four independent experiments + SEM. Statistics: 1-way ANOVA **B–D**; *p < 0.05, **p < 0.01, ****p < 0.0001; *compared to control, #compared to *Kp*OMV; ns = not significant; n = 3–4

Since recognition of LPS via TLR4 is essential for endocytosis and TRIF-activation, LPS on the OMV surface was neutralized by the lipopeptide antibiotic Polymyxin B (PB), resulting in 50% decreased Mx1 induction (Fig. 6A + B and Additional file 1: S6C), but no significant difference in NF-κB-dependent *CXCL8* transcription (Fig. 6C). Viral replication was rescued to basal level upon *Kp*OMV + PB pre-stimulation (Fig. 6D). As inhibition of LPS recognition by PB was insufficient to completely block Mx1, OMVs from endotoxin-free *Clear coli* (*Cc*) were isolated and used for pre-stimulation. *Cc* express an altered lipid A and cannot induce an activate TLR4/MD2 complex [24]. *Cc*OMVs did not induce *Mx1* (Fig. 6A + B and Additional file 1: S6C) and did not reduce IAV replication in macrophages (Fig. 6D).

It is well known that isolation of extracellular vesicles via differential ultracentrifugation results in the co-isolation of contaminants (*e.g.* proteins or bacterial cell appendages) alongside with the vesicles. In accordance with MISEV2018 guidelines [25], key experiments were repeated with OMVs isolated via a combination of ultrafiltration (UF) and size exclusion chromatography (SEC). Stimulation of THP-1 reporter cells with UF-SEC purified *Kp*OMVs resulted in induction of Mx1 (Additional file 1: Fig. S8A) and IRF reporter (Fig. S8B), while it was lost in TRIF^−/−^ macrophages (Additional file 1: Fig. S8A + B). IAV replication was blocked in *Kp*OMV pre-treated cells and rescued upon TRIF deletion (Additional file 1: Fig S8C). NF-κB reporter activity was induced by *Kp*OMVs regardless of the TRIF status of the cells (Additional file 1: Fig. S8D).

### IAV replication-inhibiting effect of OMVs is transferable to AECs

**Fig. 7:**
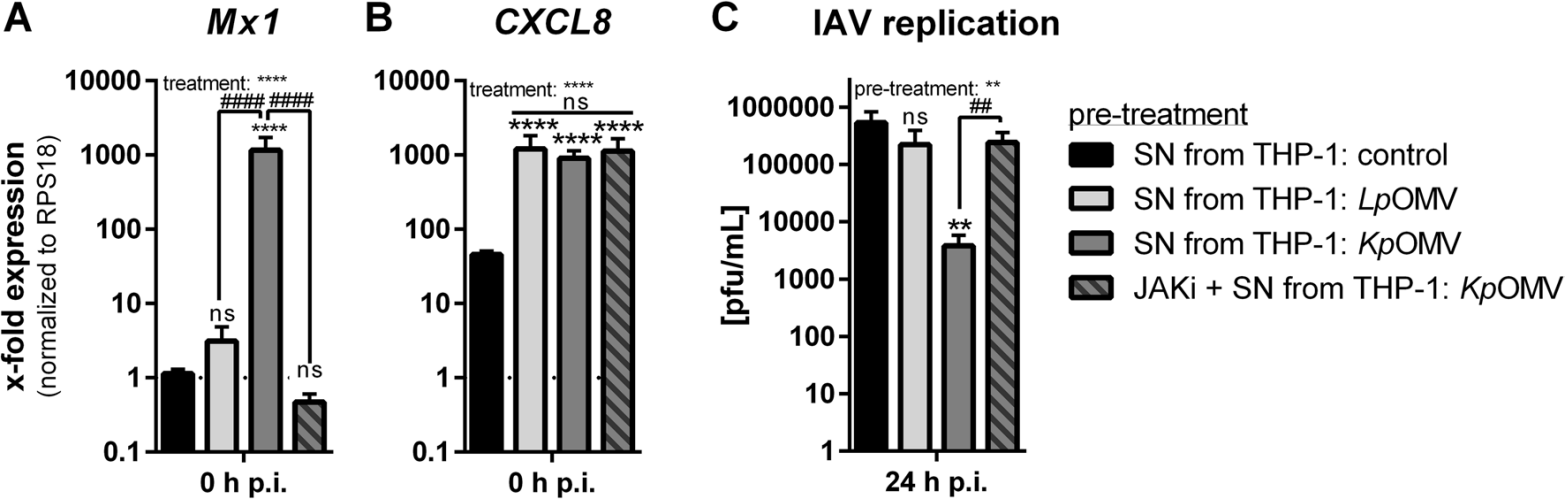
Influenza A virus replication-inhibiting effect of OMVs is transferable to AECs. THP-1 cells were incubated with *Lp*OMV or *Kp*OMV for 20 h or left untreated for control. Supernatant (SN) was sterile filtered and used for pre-stimulation of A549 cells for 20 h alone or in combination with 10 µM JAKi. **A **+ **B** *Mx1* (A) and *CXCL8* **B** expression were determined by qPCR at the time point of infection (0 h p.i.). Bars show mean values of four independent experiments + SEM normalized to untreated control cells. **C** Pre-treated A549 cells were additionally infected with influenza virus A/Hamburg/5/2009(H1N1pdm) (MOI 0.01) for 24 h. Viral replication was determined by plaque assay. Bars are mean values of four independent experiments + SEM. Statistics: 1-way ANOVA; **p < 0.01, ****p < 0.0001; * compared to control-SN; # compared to *Kp*OMV-SN; n = 4

Since macrophages are not the main cell type for IAV replication in the lung and as IFN I also acts paracrine, the transferability of the IAV replication effect to AECs was tested, as these cells, unlike macrophages, do not directly respond to OMVs/MVs (Additional file 1: Fig. S9). Supernatant (SN) from *Kp*OMV-treated macrophages induced *Mx1* in A549 cells, in contrast to *Lp*OMV-SN (Fig. 7A), while *CXCL8* was induced after both (Fig. 7B). *Kp*OMV-SN reduced viral replication (Fig. 7C), which was in line with macrophage experiments and pattern of *Mx1* induction. To confirm the dependency of the observed IAV-limiting effect on JAK/STAT-signalling, *Kp*OMV-SN was combined with JAKi, blocking *Mx1* induction upon *Kp*OMV-SN-treatment (Fig. 7A) and restoring IAV replication (Fig. 7C).

**Fig. 8:**
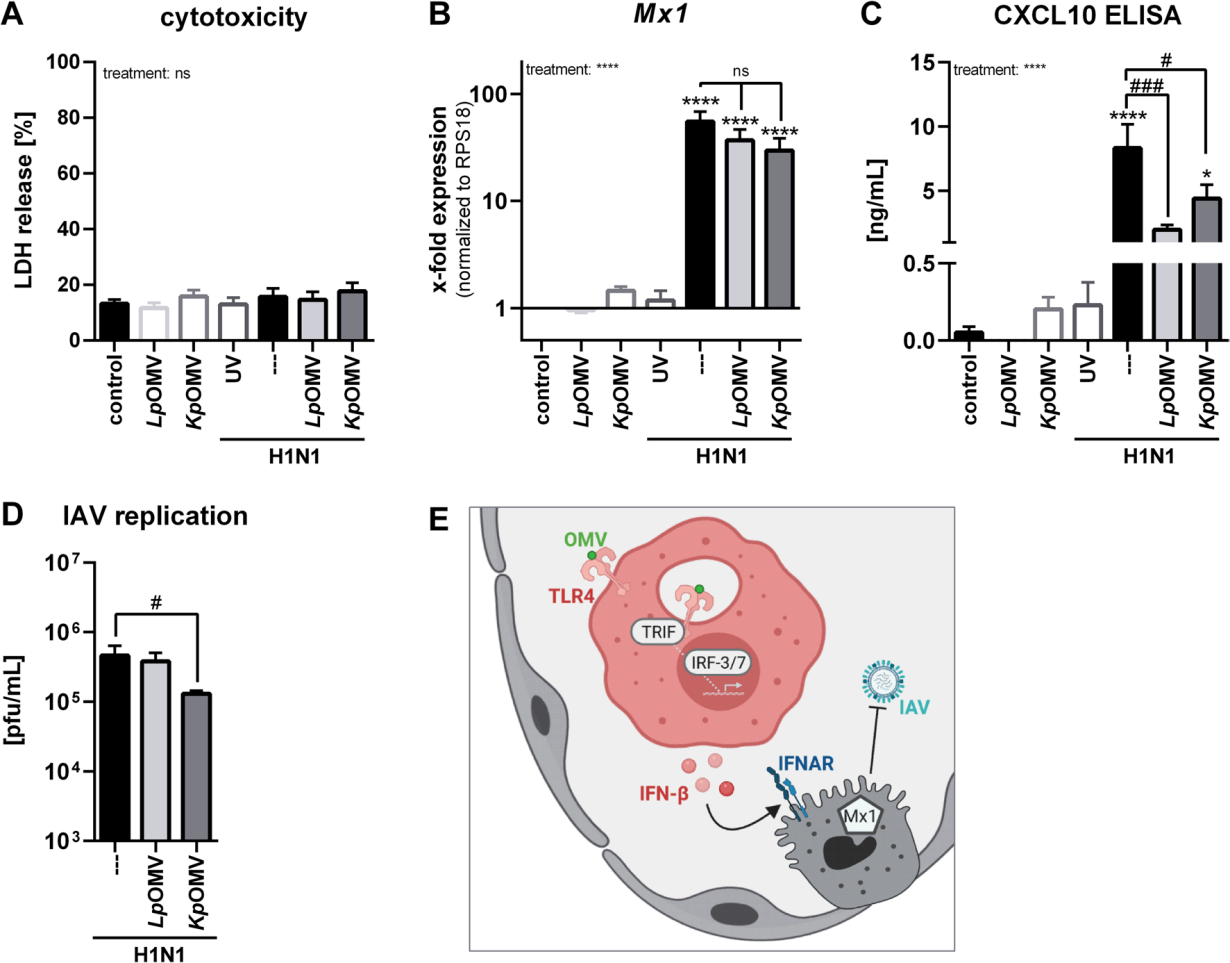
*Kp*OMV reduce influenza A virus replication in human precision-cut lung slices. Human PCLS were incubated with 1 µg/mL OMVs (*Lp*/*Kp*) for 20 h and then additionally infected with influenza A/California/04/2009(H1N1pdm) for 48 h. UV inactivation of virus served as a control. **A** Cytotoxicity was determined by quantification of released LDH from PCLS and is depicted in % compared to a total lysis. **B** *Mx1* expression was quantified by qPCR and is presented relative to *RPS18* and untreated control PCLS. **C** CXCL10 release was determined by ELISA and is depicted in ng/mL. **D** Viral replication was determined by plaque assay and is depicted in pfu/mL. Bars show mean values of three to five biological replicates + SEM. **E** Proposed model for induction of antiviral immunity of OMVs in the lung. Statistics: 1-way ANOVA (A-C), Friedman-test (D); *p < 0.05, ***p < 0.001, ****p < 0.0001; * compared to IAV infected, but not pre-treated PCLS, # as depicted in the graph; ns = not significant; n = 5

To mimic the complex regulation of immune processes in the lung, *ex* *vivo* IAV infection of human precision-cut lung slices (PCLS) was set up. *Lp*/*Kp*OMVs were not cytotoxic to the PCLS (Fig. 8A) and the combination of OMV pre-stimulation and IAV infection resulted in *Mx1* induction (Fig. 8B) and CXCL10 release (Fig. 8C). *Kp*OMVs but not *Lp*OMVs significantly reduced the IAV replication in PCLS (Fig. 8D). The observed effects were abolished by infection with UV inactivated IAV (Fig. 8A–C).

Our findings lead to a model of lung inflammation wherein AMs respond to OMVs from gram-negative bacteria with induction of IFN I, inducing antiviral responses in an autocrine manner in AMs and in a paracrine manner in AECs *in* *vitro* and in an *ex vivo* PCLS model (Fig. 8E).

## Discussion

We found that vesicles from different bacteria induce differential signalling in macrophages that is able to limit IAV replication in a subsequent infection. In line with literature, isolated OMVs from *Lp*, *Kp*, *Ec*, and *Sal*, and MVs from *Sp* were comparable in size [26–28]. Macrophages incubated with bacterial vesicles did not show cytotoxicity [29, 30], but induced a pro-inflammatory response. All vesicles triggered p38 phosphorylation and release of CXCL8 and IL-1β from primary human macrophages, while there were differences in induction of IFN I and their downstream signalling. We and others already showed that *Lp*OMVs signal via TLR2 [12, 22] and also *Sp*MVs induced a distinct activation pattern in macrophages [31]. *Lp*OMVs/*Sp*MVs activated pro-inflammatory NF-κB target genes, as described for the bacteria of origin [12, 22, 31]. Yet, recognition of *Kp*/*Ec*/*Sal*OMVs provoked, in addition to NF-κB target genes, the induction of IRF-3 phosphorylation with downstream *IFNB* expression and a long-lasting phosphorylation of STAT1 together with induction of ISGs, arguing for active IFNAR-JAK/STAT signalling. OMVs carry LPS on their surface, but the pro-inflammatory capacity of OMVs could not be mimicked with pure LPS. We and others observed that macrophages are more sensitive to OMVs compared to the same amount of pure LPS [32, 33]. OMVs contain a sum of immune agonists which are needed for the induction of a robust immune response [32]. Commercial TLR and RIG-I agonists alone or in combination with LPS could not mimic the effect. It was demonstrated before that LPS-loaded liposomes induced a more prolonged activation of TRIF-IRF-3 in macrophages compared to free LPS [34]. By using TRIF^−/−^ macrophages, we showed the dependency of ISG induction on this adaptor molecule. Moreover, endotoxin-free *Cc*OMVs or OMVs combined with LPS-masking PB were sufficient to abrogate the response of macrophages. This indicates the recognition of OMVs via TLR4 with subsequent endocytosis and TRIF-signalling. Accordingly, *Kp*/*Ec*/*Sal*OMVs were able to induce the pro-inflammatory activation of macrophages via TLR4 leading to MyD88 signalling and NF-κB dependent gene expression as well as TRIF signalling with IFN I response. This is in line with literature showing that TLR4 and TRIF together are required for immunogenicity of *Neisseria* *meningitidis* OMVs in mice [35].

As type I IFNs are master regulators of antiviral responses, we hypothesized that recognition of *Kp*/*Ec*/*Sal*OMVs affects IAV replication. We demonstrated that macrophages efficiently blocked IAV and VSV replication after TRIF-activating OMV pre-treatments, while TRIF^−/−^ rescued viral replication. To pinpoint the effect to IFNAR signalling, JAK1/2 inhibition combined with OMVs, successfully blocked STAT1-dependent *Mx1* induction and rescued viral replication in macrophages. Combination of *Kp*OMVs with PB or application of endotoxin-free *Cc*OMVs did not reduce IAV replication in macrophages as they were incapable of inducing antiviral genes.

As extracellular vesicle preparations obtained by ultracentrifugation contain free protein and bacterial cell attachments alongside with the vesicles, OMV preparations were additionally generated via a combination of ultrafiltration and size exclusion chromatography [25]. The obtained pure vesicle preparations were equally able to induce TRIF-IRF signalling with downstream Mx1 induction and blocked viral replication arguing for a direct vesicle effect.

Although AMs are the first line of defence in the lung, the majority of IAV infection and replication in a human lung takes place in AECs [36]. Therefore, AECs were stimulated with OMVs, but they did not respond to the stimulation like macrophages. Only the bronchial epithelial cell line BEAS2B responded with *CXCL8* expression, but lacked *Mx1* induction. Since we could show an antiviral gene expression depending on TLR4 and TRIF in macrophages, we hypothesize that the four tested epithelial cell lines do not express TLR4 to a similar extent as macrophages and that they failed to endocytose OMVs upon TLR4 activation. This is in line with literature showing that isolated human AECs do not respond to LPS as paired AMs did [37]. As we linked the antiviral status upon OMV pre-incubation to IFN I, we used supernatant of OMV-stimulated macrophages to pre-stimulate AECs. The conditioned medium of *Kp*OMV-stimulated macrophages, unlike direct vesicle stimulation, induced *Mx1* expression in these cells and reduced IAV replication in subsequent infection experiments. Supernatant from *Lp*OMV treated macrophages induced *CXCL8* in epithelial cells, but caused no *Mx1* induction and changes in viral replication. To attribute the effect observed with conditioned media to IFN I, supernatant was combined with a JAK1/2 inhibitor, blocking *Mx1* induction and had no effect on viral replication. Since the interplay of the different cell types in a human lung is more complex and cannot fully be mimicked by conditioned media, we extended our approach to viable sections of human distal lung tissue, which has been widely used for studies on host–pathogen interactions including influenza virus and bacterial infections [38–41]. Contrasting the used in vitro models, the ex vivo human lung tissue maintains the three-dimensional architecture of the lung and allows a physiological interplay among the resident cell types in the lung, yet it lacks the possibility of influx of further recruited immune cells. Consecutive infection of *Kp*OMV-stimulated PCLS with IAV decreased the viral load compared to not pre-treated controls. As all the AECs tested in vitro did not respond to direct bacterial vesicle stimulation, we hypothesise that in this ex vivo model the AMs are the predominant responding cell type. Since an influenza isolate was chosen for the subsequent infection that exclusively infects and replicates in AECs, it can be presumed that this is a paracrine antiviral effect originating from AMs and transmitted to AECs.

Based on the data obtained here, we propose the following model (Fig. 8E): OMVs from gram-negative bacteria can activate human macrophages in a classical pro-inflammatory manner and anti-virally prime them via TLR4 and TRIF after successful endocytosis of the vesicles. IFN I release can in turn render macrophages and/or AECs antiviral via IFNAR and JAK/STAT signalling. Subsequent infection of these cells causes an Mx1-mediated decrease in viral load. Since we did not have access to primary human AMs, the experiments were performed using human BDMs as a model. Considering the different developmental origin and priming of these macrophages, it is conceivable that AMs would have shown a weaker pro-inflammatory response in contrast to BDMs. Bacterial vesicles are already used in vaccination strategies against different pneumonia-inducing pathogens (reviewed in [42]). Hence, OMVs do not only represent a tool for potential systemic vaccination strategies against their host bacteria, but could also be used locally to combat viral infections by activating resident innate immune cells. Future *in* *vivo* studies are needed to test whether these *in* *vitro* findings are applicable to other viral pathogens. In addition, it needs to be noted that a possibly occurring endotoxic response must be critically monitored to avoid exaggerated immune responses in vivo. The induction of type I IFN needs to be tightly controlled, as it was shown in healthy individuals that inhalation of a TLR7 agonist was initially well tolerated after the first dose, but led to an increased TNF-α and IFN I response and influenza-like symptoms, after a second dose [43]. To combat adverse effects, genetically modified versions of OMVs may be needed, inducing a balanced immune response and achieving a good applicability.

Taken together, we herein present a model of how OMVs can induce antiviral signalling in human macrophages and how this can be used to prevent IAV replication.

## Supplementary Information

**Additional file 1** Supplementary Figures.
