# Supplementary material for: Bacterial vesicles block viral replication in macrophages via TLR4-TRIF-axis
Source: Cell Commun Signal. 2023 Mar 28;21:65. doi: 10.1186/s12964-023-01086-4 (PMC10045439; doi:10.1186/s12964-023-01086-4)
Supplement: Supplementary file 2 — Additional file 1 Supplementary Figures. [file 12964_2023_1086_MOESM2_ESM.docx]

**Supplementary Figures**

**Figure S1:**


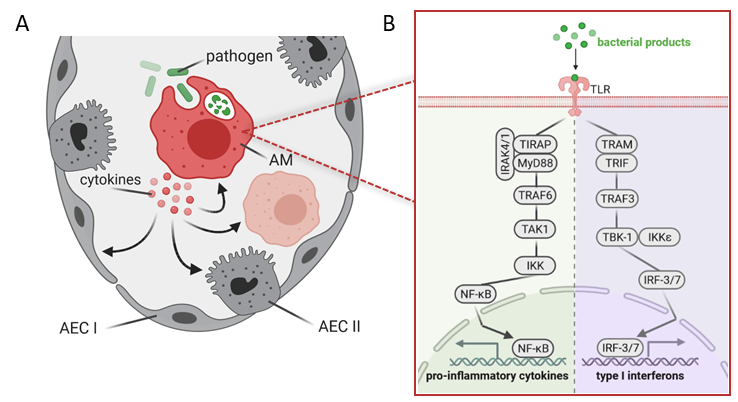


**Figure S1: Schematic illustration of alveolar macrophage function and intracellular signaling.** (A) Schematic illustration of an alveolus with an alveolar macrophage (AM) responding to an invading bacterial pathogen. Released cytokines act in an auto- and/or paracrine manner on surrounding alveolar epithelial cells (AEC I + II) and AMs. (B) Bacterial products activate AMs via TLR engagement. Signaling can occur via MyD88 and NF-κB or TRIF and IRF-3/7, which culminate in the transcription of distinct subsets of inflammatory genes.

**Figure S2:**

**Figure S2: Bacterial vesicles dosage and cytotoxicity in macrophages.** (A) Mean OMVs/MVs sizes are depicted in nm. Mean values +SEM of three different vesicle preparations are shown. (B) Vesicle concentration of three different vesicle preparations per bacteria are depicted. Bars represent mean values +SEM. (C) Calculation of applied vesicle dosage based on the protein concentration of OMVs/MVs preparations and particle concentration from nFCM measurement. Mean values of three independent vesicle preparations +SEM are shown. (D) BDMs were stimulated with OMVs/MVs (1 µg/mL each) from different bacteria or left untreated as control. Cytotoxicity was determined by quantification of released LDH from cells and is depicted in % compared to a total lysis. Mean values +SEM of four biological replicates are shown. Statistics: 1-way ANOVA (A-C), 2-way-ANOVA (B); ns=not significant; n=3-4.

**Figure S3:**





**Figure S3: Characterization of response of human macrophages to bacterial vesicles.** (A-B) BDMs were stimulated with OMVs/MVs (1 µg/mL each) from different bacteria or left untreated for control for up to 48 h. (A) *CXCL8* expression was determined by qPCR, results are normalized to *RPS18* and are depicted relative to untreated control cells. (B) IL-1β release was determined by ELISA and is depicted in pg/mL. Bars represent mean values +SEM from three to four independent experiments. Statistics: 2-way ANOVA; **p<0.01, ***p<0.001, ****p<0.0001; n=3-4.

**Figure S4:**





**Figure S4: Quantification of Western Blots.** (A-D) from Figure 1G. (E) from Figure 2D. (F+G) from Figure 2G. Bars represent mean values +SEM from three to four independent experiments. Statistics: 1-way ANOVA; *p<0.05, **p<0.01, ***p<0.001, ****p<0.0001; n=3-4.

**Figure S5:**


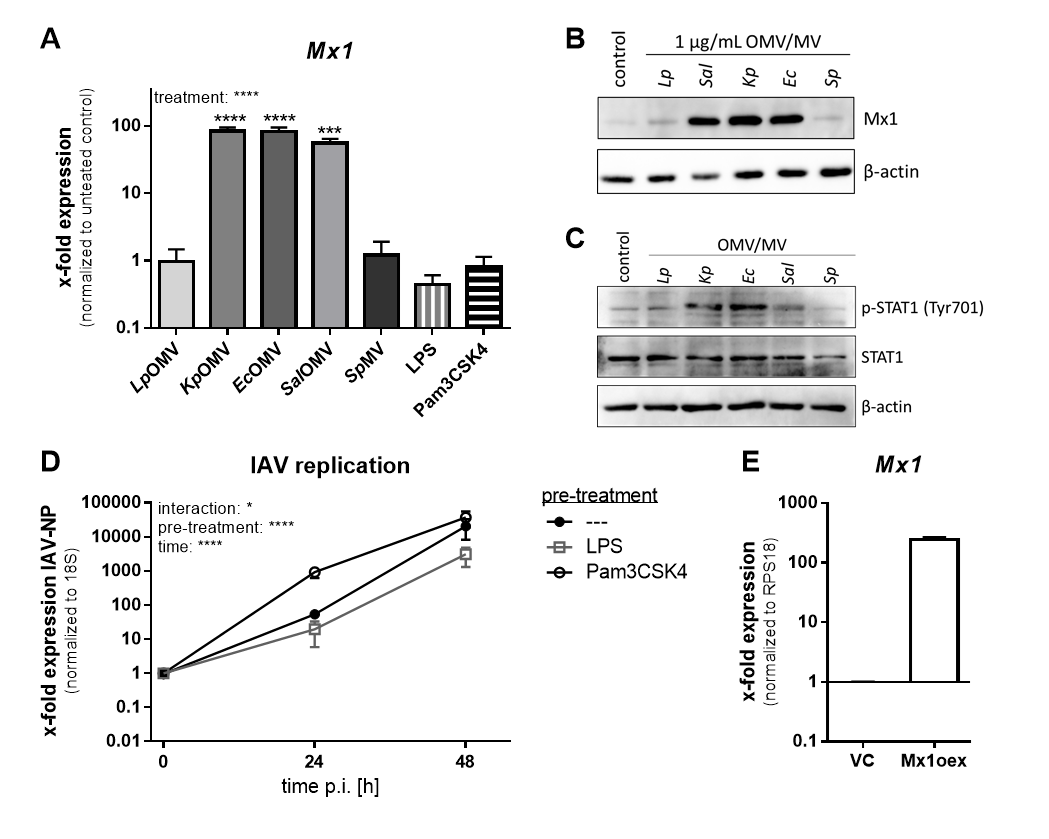


**Figure S5: IAV replication in macrophages.** (A-C) PMA-differentiated THP-1 cells were stimulated with OMVs/MVs (1 µg/mL each) from different bacteria, LPS (1 µg/mL) or Pam3CSK4 (200 ng/mL) or left untreated as control for 20 h. (A) Expression of *Mx1* was measured by qPCR, results are normalized to *RPS18* and are depicted relative to untreated control cells. Bars represent mean values +SEM from four independent experiments. (B+C) Expression of Mx1 (B) and phosphorylation (Tyr701) and expression of STAT1 (C) was determined by Western Blot. Representative result of three to four biological independent replicates is shown. (D) After pre-treatment, cells were infected with A/WSN/33(H1N1) (MOI 0.001) for 24 and 48 h. IAV replication was determined by qPCR against IAV-NP normalized to 18S. Mean values ±SEM of four independent experiments are shown. (E) Overexpression of Mx1 in THP-1 cells compared to corresponding empty vector control (VC) cells determined by qPCR. Statistics: 1-way ANOVA (A) or 2-way ANOVA (D); *p<0.05, ***p<0.001, ****p<0.0001; n=4.

**Figure S6**





**Figure S6: Quantification of Western Blots.** (A) from Figure 4D. (B) from Figure 5B. (C) from Figure 6A. Bars represent mean values +SEM from three to four independent experiments. Statistics: A: 2-way ANOVA; * compared to DMSO, # compared to *Kp*OMV or *Sal*OMV; B: 2-way ANOVA; * compared to unstimulated Dual control, # as depicted in the graph; C: 1-way ANOVA; * compared to control, # compared to *Kp*OMV; *p<0.05, **p<0.01, ***p<0.001, ****p<0.0001; n=3-4.

**Figure S7:**


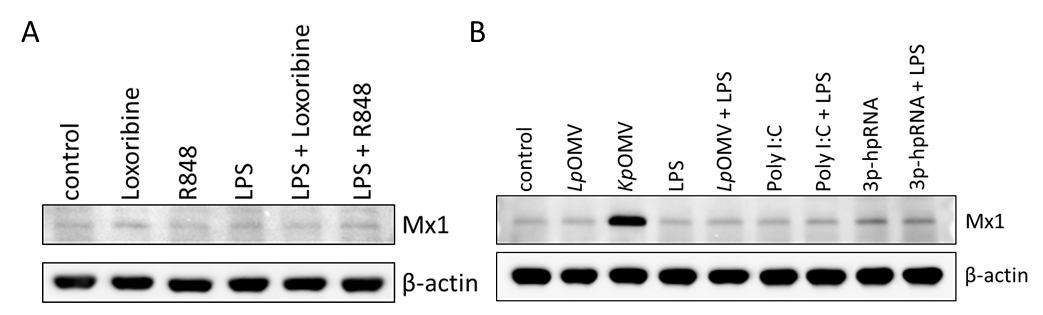


**Figure S7: Induction of Mx1 with commercial immune agonists.** THP-1 cells were incubated for 20 h with different immune agonists or *Lp*/*Kp*OMV (1 µg/mL) alone or in combination (A+B). Mx1 protein expression was analyzed by Western Blot. Representative results of three biological independent experiments are shown.

**Figure S8:**

**
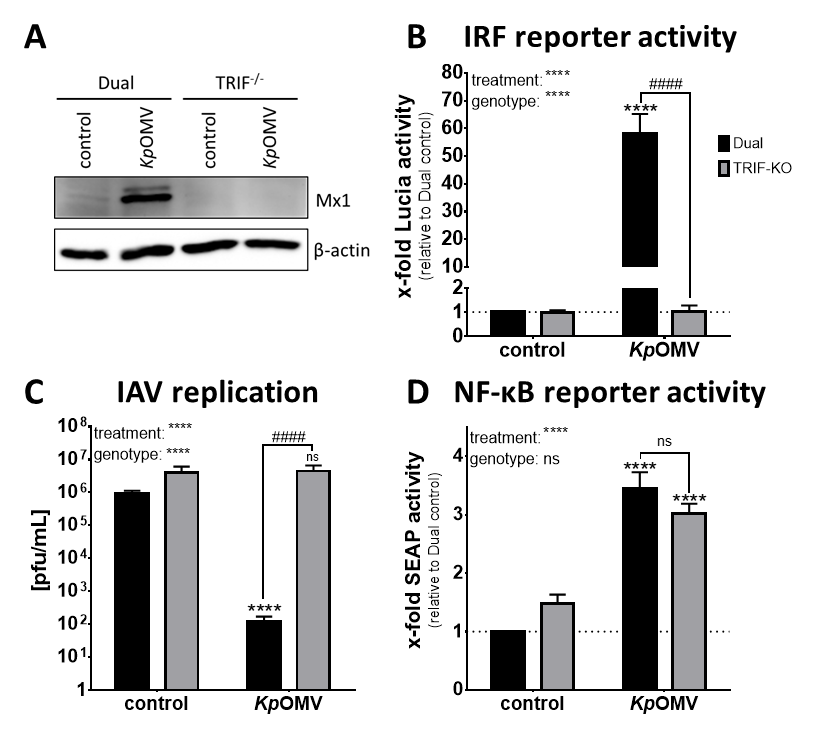
**

**Figure S8: UF-SEC purified OMVs provoke TRIF-dependent pro-inflammatory response.** THP-1 reporter cells (=Dual; black bars) and TRIF^-/-^ cells (grey bars) were differentiated and subsequently stimulated with *Kp*OMV (multiplicity of vesicles: 1,000) for 20 h or left untreated for control. After 20 h, supernatant or proteins were collected. (A) Representative Western Blot image of Mx1 protein expression. (B+D) Lucia reporter activity (B) and SEAP reporter activity (D) was determined in cell culture supernatant. The same supernatant was used to determine the activity of both reporters. (C) *Kp*OMV pre-stimulated cells (Dual and TRIF^-/-^) were additionally infected with A/WSN/33(H1N1) (MOI 0.1) for 24 h. Bars show mean values of four (C+D) to six (B) independent experiments +SEM. Statistics: 2-way ANOVA (B-D); ****p<0.0001; * compared to unstimulated Dual control, # compared to *Kp*OMV stimulated Dual cells; ns=not significant; n=4-6.

**Figure S9:**


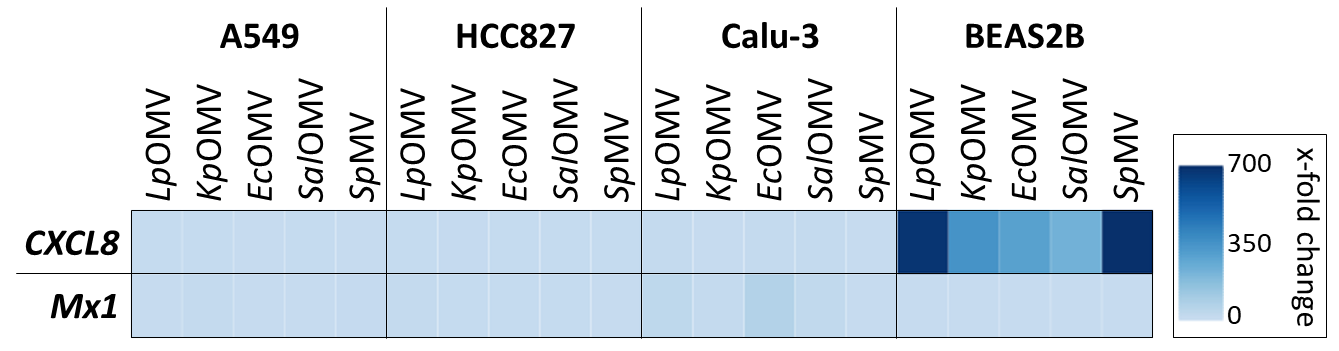


**Figure S9: Pro-inflammatory response of epithelial cells to OMVs/MVs.** Different alveolar and bronchial epithelial cell lines (from left to right: A549, HCC827, Calu-3, BEAS2B) were incubated with OMVs/MVs (*Lp*, *Kp*, *Ec*, *Sal*, *Sp*; 1 µg/mL each) for 20 h. Relative expression of *CXCL8* (upper row) and *Mx1* (lower row) was determined. n=2-4.
